# Methodological and statistical errors distort the effects of glucagon-like peptide-1 receptor agonist drugs on body composition in patients with type 2 diabetes mellitus

**DOI:** 10.1186/s13098-025-02051-6

**Published:** 2026-01-20

**Authors:** Eric T. Trexler

**Affiliations:** Department of Health, Wellness and Physical Education, Duke University, Durham, USA

**Keywords:** Glucagon-like peptide 1 receptor agonist, Meta-analysis, Sarcopenia, Skeletal muscle mass, Type 2 diabetes

## Abstract

In August of 2025, Wang and colleagues published a systematic review and meta-analysis quantifying the effects of glucagon-like peptide-1 receptor agonist (GLP1RA) drugs on skeletal muscle mass in patients with type 2 diabetes mellitus. This paper is of considerable clinical importance, as sarcopenia, obesity, and diabetes are prevalent conditions of high public health interest. Despite the clinical importance of this meta-analysis, its effect estimates and conclusions are distorted by several common meta-analytic errors. A preliminary check of the article revealed errors across several steps of the meta-analytic process. The present correspondence identifies and describes specific errors related to study inclusion, risk of bias assessment, data extraction, analysis, and interpretation. In some instances, errors were severe enough to flip the conclusions derived from statistical tests. The present correspondence does not purport to identify every error in the meta-analysis by Wang and colleagues, but presents objective evidence to raise concerns that warrant clarification and correction.

 In August of 2025, Wang and colleagues published a systematic review and meta-analysis quantifying the effects of glucagon-like peptide-1 receptor agonist (GLP-1 RA) drugs on skeletal muscle mass in patients with type 2 diabetes mellitus [1]. This paper is of considerable clinical importance, as sarcopenia, obesity, and diabetes are prevalent conditions of high public health interest. Despite the clinical importance of this meta-analysis, its effect estimates and conclusions are distorted by several common meta-analytic errors. There are also apparent errors in the step of data extraction. For example, the body weight values extracted from Kondo et al. [2] presented in Fig. 3A of the meta-analysis (65.9 ± 2.9 and 62.6 ± 2.8) mistakenly list standard error values in place of standard deviation values. This common error shrinks the denominator of the effect size calculation, leading to inflated effect sizes.

A preliminary check of the article revealed errors across several steps of the meta-analytic process. The text indicates that only randomized controlled trials were eligible for inclusion. However, the included study by Keskin and Yaprak [3] is a retrospective analysis of medical records of previously treated patients. While Keskin and Yaprak clearly described their study as a retrospective analysis and did not use the words “random,” randomised,” or “randomized” in the text of their article, Wang and colleagues rated its randomization procedures as having low risk of bias. As such, there are errors in the steps of study inclusion and risk of bias assessment.

Wang and colleagues acknowledge that the weight values extracted from the Kondo et al. study (65.9 ± 2.9 and 62.6 ± 2.8) are unadjusted post-test values. In this study, the baseline weight difference between groups was considerably larger than the effect of either intervention. By using unadjusted post-test values instead of the change scores that were reported in the text, the direction of treatment effect has been erroneously flipped. While the authors acknowledge this in the manuscript, acknowledgement does not sufficiently address the underlying issue. It is statistically defensible to include a mixture of change score and post-test scores in a meta-analysis when the effect measure is (unstandardized) mean difference [4]. Ideally, Wang and colleagues would provide a less biased estimate of the pooled mean difference by carefully evaluating the suitability of post-test values based on similarity of baseline values between groups. In cases where randomization fails to yield groups with approximately identical baseline values, it is advisable to enter the change score into the meta-analysis in place of the post-test value.

To further explore the results of the meta-analysis by Wang et al., I extracted values from a subsample of forest plots (Figs. 3 and 4, and 5) and attempted to reproduce the analyses. I was able to successfully reproduce the results presented in Fig. 3B, 4A, 4B, and 5C. Initially I was unable to reproduce the results presented in Figs. 3A, 5A, and 5B. I then identified the source of error, which relates to confidence interval adjustments. Wang et al. indicate that their analysis applied the Hartung-Knapp adjustment with ad hoc modification, and that analyses were completed using the *metafor* package in R software. For calculation of confidence intervals, the default method in *metafor* assumes a standard normal distribution. Other options include use of the Hartung-Knapp method or, alternatively, a variation of the Hartung-Knapp method that uses an ad hoc modification to ensure that the value of the scaling factor is always 1 or greater. The former is known as the Hartung-Knapp method, whereas the latter is known as the Hartung-Knapp method with ad hoc modification.

The analyses used to produce Fig. 3B, 4A, 4B, and 5C applied the Hartung-Knapp method to calculate 95% confidence intervals. Analyses producing Fig. 3B C are compatible with the Hartung-Knapp method with ad hoc modification, but Figs. 4A and 4B are not. The analyses used to produce Figs. 3A, 5A, and 5B did not apply any variation of the Hartung-Knapp method, instead using the default method. Table 1 presents the pooled effect estimates (and 95% confidence intervals) for these analyses using three separate analytical approaches: the default method, the Hartung-Knapp method, and the Hartung-Knapp method with ad hoc modification. For this subset of seven analyses, three appear to be calculated using the default method and four appear to be calculated using Hartung-Knapp method. Using the Hartung-Knapp method with ad hoc modification, as stated in the methods, would only allow successful replication of the reported results in the special cases in which the Hartung-Knapp method and the Hartung-Knapp method with ad hoc modification happen to yield identical confidence intervals.

**Table 1:** Pooled effect estimates for Figs. 3, 4 and 5 using three different methods for confidence interval calculation

Dataset	Method	k	MD	95% CI Lower	95% CI Upper	*P* value
Figure 3A	adhoc	3	4.611	−14.717	23.940	0.4126
Figure 3B*	**adhoc**	**3**	**0.585**	**−0.685**	**1.855**	**0.1859**
Figure 4A	adhoc	6	−2.551	−7.206	2.105	0.2180
Figure 4B	adhoc	5	−1.826	−3.936	0.283	0.0740
Figure 5A*	adhoc	2	−8.001	−33.445	17.444	0.1561
Figure 5B	adhoc	2	−2.757	−8.517	3.003	0.1038
Figure 5C*	**adhoc**	**3**	**0.736**	**−54.926**	**56.398**	**0.9598**
**Figure 3A**	**default (z)**	**3**	**4.611**	**−4.194**	**13.416**	**0.3047**
Figure 3B	default (z)	3	0.585	0.037	1.133	0.0365
Figure 4A	default (z)	6	−2.551	−6.101	0.999	0.1590
Figure 4B	default (z)	5	−1.826	−3.315	−0.337	0.0162
**Figure 5A**	**default (z)**	**2**	**−8.001**	**−11.925**	**−4.076**	**0.0001**
**Figure 5B**	**default (z)**	**2**	**−2.757**	**−3.645**	**−1.868**	**0.0000**
Figure 5C	default (z)	3	0.736	−22.692	24.164	0.9509
Figure 3A	knha	3	4.611	−14.620	23.842	0.4107
Figure 3B*	**knha**	**3**	**0.585**	**−0.685**	**1.855**	**0.1859**
**Figure 4A**	**knha**	**6**	**−2.551**	**−6.992**	**1.890**	**0.1998**
**Figure 4B**	**knha**	**5**	**−1.826**	**−3.883**	**0.231**	**0.0693**
Figure 5A*	knha	2	−8.001	−33.445	17.444	0.1561
Figure 5B	knha	2	−2.757	−7.112	1.598	0.0787
Figure 5C*	**knha**	**3**	**0.736**	**−54.926**	**56.398**	**0.9598**

MD, mean difference; CI, confidence interval. Rows with bold text match the results reported in the original manuscript by Wang et al.

*Denotes analyses for which Hartung-Knapp method (knha) and Hartung-Knapp method with ad hoc modification (adhoc) yield identical results due to sufficiently large scaling factor

The meta-analysis by Wang et al. also contains interpretation errors when discussing pooled mean difference (MD) values for body mass index (BMI), fat mass (FM), body fat ratio (BFR), and visceral fat area (VFA). The authors state “Compared to the control group, patients who received GLP-1 RA experienced significant weight loss. (MD = −2.55, [−6.99; 1.89], *P* = 0.20).” The statement implies statistical significance, which conflicts with the stated *P* value of 0.20, which appears to be calculated using the Hartung-Knapp method (with no ad hoc modification). The authors state “The results showed that GLP-1 RA caused statistically significant decrease in BMI compared with other treatments (MD = −1.83, [−3.88; 0.23], *P* < 0.05).” Upon recalculation, this *P* value is 0.069 (greater than 0.05) using the Hartung-Knapp method, and even higher if using the ad hoc modification (Table 1). The authors state “Compared to other hypoglycemic drugs, GLP-1 RA were found to significantly lower FM (MD=−8.0, [−11.93; −4.08], *P* < 0.01).” Upon recalculation, this *P* value is 0.1561 (greater than 0.05) using the Hartung-Knapp method, with or without ad hoc modification. The authors state “There was a significant reduction in BFR with GLP-1 RA compared to the control group (MD=−2.76, [−3.65; −1.87], *P* < 0.01).” Upon recalculation, this *P* value is 0.0787 (greater than 0.05) using the Hartung-Knapp method, and even higher if using the ad hoc modification (Table 1). In addition, the authors state “There were significant differences in VFA for GLP-1 receptor agonists compared to other drugs (MD = 0.74, [−54.93; 56.40], *P* < 0.05).” The *P* value for this comparison was at least 0.95 using all three methods (Table 1).

In summary, the recent meta-analysis by Wang et al. contains errors related to study inclusion, risk of bias assessment, data extraction, analysis, and interpretation. In many instances, errors were severe enough to flip the conclusions derived from statistical tests. It is possible that there are substantive justifications for some or all of these impactful analytic choices, but the text provides no acknowledgement or explanation for these deviations from the stated analytical approach. The present commentary does not purport to identify every error in the meta-analysis by Wang and colleagues, but presents objective evidence to raise considerable concerns that warrant clarification and correction.

## Data Availability

All data and code used for this reanalysis are available in the Open Science Framework repository. (https://osf.io/rzs43/overview?view_only=98a754507a474637b4090d21e2f3fdfb).

## Abbreviations

GLP1RA: Glucagon like peptide-1 receptor agonist
MD: Mean difference
CI: Confidence interval
BMI: Body mass index
FM: Fat mass
BFR: Body fat ratio
VFA: Visceral fat area
